# The influence of patient race on the use of diagnostic imaging in United States emergency departments: data from the National Hospital Ambulatory Medical Care survey

**DOI:** 10.1186/s12913-020-05698-1

**Published:** 2020-09-07

**Authors:** Andrew B. Ross, Vivek Kalia, Brian Y. Chan, Geng Li

**Affiliations:** 1 Department of Radiology, School of Medicine and Public Health, University of Wisconsin, 600 Highland Ave, Madison, WI 53792 USA; 2 Department of Radiology, University of Michigan Health System, 1500 E Medical Center Dr, Ann Arbor, MI 48109 USA; 3 Department of Radiology, School of Medicine, University of Utah, 30 N. 1900 E., Salt Lake City, UT 84132 USA; 4 Department of Biostatistics and Medical Informatics, School of Medicine and Public Health, University of Wisconsin, 600 Highland Ave, Madison, WI 53792 USA

**Keywords:** Race, Ethnicity, Diagnostic imaging, Bias, Emergency services, Health services

## Abstract

**Background:**

An established body of literature has shown evidence of implicit bias in the health care system on the basis of patient race and ethnicity that contributes to well documented disparities in outcomes. However, little is known about the influence of patient race and ethnicity on the decision to order diagnostic radiology exams in the acute care setting. This study examines the role of patient race and ethnicity on the likelihood of diagnostic imaging exams being ordered during United States emergency department encounters.

**Methods:**

Publicly available data from the National Hospital Ambulatory Medical Care Survey Emergency Department sample for the years 2006–2016 was compiled. The proportion of patient encounters where diagnostic imaging was ordered was tabulated by race/ethnicity, sub-divided by imaging modality. A multivariable logistic regression model was used to evaluate the influence of patient race/ethnicity on the ordering of diagnostic imaging controlling for other patient and hospital characteristics. Survey weighting variables were used to formulate national-level estimates.

**Results:**

Using the weighted data, an average of 131,558,553 patient encounters were included each year for the 11-year study period. Imaging was used at 46% of all visits although this varied significantly by patient race and ethnicity with white patients receiving medical imaging at 49% of visits and non-white patients at 41% of visits (*p* < 0.001). This effect persisted in the controlled regression model and across all imaging modalities with the exception of ultrasound. Other factors with a significant influence on imaging use included patient age, gender, insurance status, number of co-morbidities, hospital setting (urban vs non-urban) and hospital region. There was no evidence to suggest that the disparate use of imaging by patient race and ethnicity changed over the 11-year study time period.

**Conclusion:**

The likelihood that a diagnostic imaging exam will be ordered during United States emergency department encounters differs significantly by patient race and ethnicity even when controlling for other patient and hospital characteristics. Further work must be done to understand and mitigate what may represent systematic bias and ensure equitable use of health care resources.

## Background

Despite significant attention and attempts at systemic change, racial and ethnic related inequalities in the provision of health care remain a significant public health problem in the United States [1–3]. These disparities persist across the full range of health care settings and are not fully explained by differences in socio-economic status, culture, patient preferences, or racial variation in disease severity [4, 5]. The body of previous work on this topic continues to show concern for systematic bias within the health care system towards minority racial and ethnic groups [6, 7]. Although bias may be implicit, the effects are pervasive and may influence diagnostic decision making. For example, previous studies have shown that minority groups may receive fewer diagnostic tests when evaluated for pediatric acute gastroenteritis or mild traumatic brain injury, may be less likely to be referred for screening mammography, and may be less likely to receive necessary cardiac procedures [8–12].

Although differential health care outcomes occur in all health care settings, the problem may be particularly acute in the Emergency Department (ED) setting which may serve as a de facto source of primary care for disadvantaged groups and where a large number of care decisions must be made, often with limited time and resources [13–15]. When seen in the ED, minority groups may be triaged to a lower acuity than white patients with similar conditions, are less likely to receive pain medication, are less likely to be admitted to an inpatient service, and face longer wait times than white patients [16–21]. As the ED often serves as a gateway to the health care system, understanding health care disparities inherent to the ED setting may be of particular importance for advancing health equity.

Diagnostic decision making in the ED is a complex, multi-faceted process. The physician must rapidly assess the patient with a history and physical examination and decide what—if any—further diagnostic testing should be performed. This may include the ordering of diagnostic imaging exams such as x-rays, computed tomography (CT) scans, magnetic resonance imaging (MRI), or ultrasound. Although guidelines may exist for specific patient presentations [22–24], it is often up to the individual physician to decide how to proceed. This subjective assessment process is subject to potential bias that may lead to differential care for minority groups.

Although diagnostic imaging is not indicated for every patient encounter in the ED, medical imaging is a fundamental and measurable component of the diagnostic process. Understanding racial and ethnic based differences in its use is of primary importance but may also serve as a marker for broader inequity during diagnostic decision making. To date, there has been little research on the role of race and ethnicity on imaging utilization during the patient evaluation process in the acute care setting. Previous research has suggested that minority groups may be less likely to receive diagnostic imaging in the ED [25] although the extent to which this occurs on a national level and how this trend has changed over time remains poorly understood. The aim of our study is to build on this previous research by using survey weighted data from a large nationally representative data set to evaluate the influence of patient race/ethnicity on the decision to order diagnostic imaging exams in the ED. Our hypothesis is that the decision to order diagnostic imaging may be subject to implicit bias leading to lower imaging utilization rates in racial and ethnic minority groups. Additionally, trends in imaging use over time are visually and quantitatively explored to assess whether the extent of any racial/ethnic bias has changed over the study time period of 2006–2016. Additional patient and hospital factors that may influence the use of diagnostic imaging are explored to put the issue in context.

## Methods

### Data source

The National Hospital Ambulatory Medical Care Survey (NHAMCS) is a nationally representative database of United States outpatient visits administered annually and designed by the National Center for Health Statistics to provide information on the utilization of health care services in the hospital-based outpatient environment. A waiver of exemption was provided by the University of Wisconsin School of Medicine and Public Health institutional review board for the use of this publicly available anonymized data set. The Emergency Department (ED) sample focuses on provision of care within the ED setting. The data set uses a four-stage probability sampling design. The primary sampling unit is geographical area further stratified by hospital, ED unit, and finally patient visits. The complex survey design with stratification, clustering, and patient visit weighting variables allows for national level estimates of outcomes although only a smaller subset of hospitals is included in the survey. Participating hospitals are randomly assigned to one of 13 four-week survey periods occurring annually. The sample frame includes hospitals in all 50 states and the District of Columbia excluding federal, military, and Veteran’s Administration facilities. Survey sites include a distribution of both urban and rural hospitals, regional trauma centers, and smaller non-trauma sites representing the full range of ED settings and services. It should be noted that the data reflect patient visits, not individual patients, and therefore patients who return more than once during the 4-week sampling period may be represented in the dataset multiple times. Data are centrally processed and validated both manually and by computer algorithm for consistency. The full data collection methodology for the NHAMCS is described on the National Center for Health Statistics website where the data used in this study are publicly available [26]. Data from the 2006–2016 survey years were combined for analysis in this study.

### Study population

Because this study seeks to broadly investigate the differential use of imaging by patient race/ethnicity for all types of patient encounters, the study sample consisted of all emergency department encounters in the data set for the included years (2006–2016). The unweighted data include 325,037 ED visits over the 11 years included in the analysis. As the survey reflects patient visits, not individual patients this may reflect a small number of repeat visits from the same patients during the 4-week sampling time period at any given sample site. With survey weighting, this provides estimates on an average of 131,558,553 patient encounters each year for the 11-year study period.

### Study variables

#### Outcome variables

The primary outcome variable for this study was the ordering of any diagnostic imaging examination of any modality including x-ray, CT, MRI, or US. These imaging categories were also evaluated independently to analyze the use of imaging by specific modality.

#### Structured variables

As the aim of the study was to evaluate the influence of patient race/ethnicity on the likelihood of diagnostic imaging use, patient race and ethnicity, was the primary predictor for the outcome variable. For the NHAMCS, information on patient race and ethnicity is completed by hospital staff using a standardized patient record form [27]. Survey instructions state that this should be, “based on observation or the hospital’s usual practice or knowledge” [28]. Racial categories include white, black, Asian/Pacific Islander, American Indian/Eskimo/Aleut. Ethnic categories included Hispanic and non-Hispanic. Missing data for non-responders in these categories is imputed. Our analysis included the imputed values for missing responses in the data set concordant with previous NHAMCS publications and as recommended for this type of modeling [29–31]. For our analysis, the combined survey variable for race and ethnicity was used with the collapsed categories of white (non-Hispanic), black (non-Hispanic), Hispanic, and Asian/other to allow for sufficient numbers within groups for meaningful analysis. Additional variables used in the analysis included the demographic variables of age and gender; patient characteristics including expected primary payor (private, Medicaid, Medicare, self-pay/unknown/other), and total number of co-morbid diseases; as well as hospital characteristics including U.S. region (Northeast, Midwest, South, and West), and setting (urban versus non-urban).

### Statistical analysis

Demographic variables for the study sample of U.S. ED visits were tabulated by race/ethnicity including age, gender, primary payor, and the proportion of ED visits at which a diagnostic imaging exam was ordered both for any imaging exam and further subcategorized by modality (x-ray, ultrasound, magnetic resonance imaging, or computed tomography). Analysis was performed to evaluate whether these variables differed significantly across racial/ethnic groups including an ANOVA test for patient age and total number of chronic conditions and a Pearson’s chi-squared test to compare patient gender, distribution of insurance status, and proportion of different types of imaging exams ordered. A Bonferroni correction was used to allow for multiple comparisons in this portion of the analysis with a corrected significance level of *p* < 0.005. To incorporate the complex survey design, the stratification, clustering, and weighting variables provided in the data set were utilized to provide weighted counts and proportions. This allows for national level estimates that are generalizable to all United States ED visits.

To illustrate imaging utilization rates by race/ethnicity over the study time period, the proportion of ED visits with imaging use was graphed by year to compare utilization between whites and non-white minority groups and to compare utilization within non-white minority sub-groups.

For the regression analysis, the dependent variable was whether any diagnostic imaging exam was ordered during the patient encounter—coded as a dichotomous yes or no—with additional regression models performed for each individual imaging modality including x-ray, CT, MRI, and US. The independent variable was patient race/ethnicity classified in the survey as white (non-Hispanic), black (non-Hispanic), Hispanic, Asian/other. In keeping with our hypothesis that minority groups may be less likely to have diagnostic imaging ordered during an encounter, white patients were set as the reference group for the regression analysis. Covariates for control in the regression model included other available patient and hospital level characteristics that might reasonably influence whether an imaging exam was ordered. Selection of these variables was based on similar analyses investigating racial/ethnic bias in the ED setting [18] with the intention of controlling for disease severity and socio-economic influencers. These included patient age, gender, primary insurance payor, total number of comorbidities, hospital region, hospital setting (urban or non-urban), and survey year. An interaction term of race by survey year was included in the model to assess whether the influence of race on image ordering changed during the 11-year study period. Odds ratios for the likelihood of diagnostic imaging use were constructed for white patients versus all non-white minority racial/ethnic groups, and pair-wise comparisons were performed between white patients and each individual minority group to compare overall imaging use and by specific imaging modality (x-ray, CT, MRI, ultrasound). The regression analysis used the weighted data to allow for the complex survey design. Analysis was performed in SAS version 9.4 (SAS Institute Inc., Cary, NC, USA).

## Results

Demographic characteristics of patients seen during these ED visits are tabulated by race in Table 1. Compared to whites, non-white minority patients were significantly younger. The most common payor for white patients was private insurance whereas Medicaid was most common for patients in minority groups. There were also small but statistically significant differences in gender distribution and total number of co-morbid chronic conditions. Between racial/ethnic groups, there were significant differences in the proportion of visits at which imaging was ordered. Across patients of all races, imaging was ordered during 46% of visits. However, this differed significantly by race as shown in Table 1, with white patients receiving medical imaging at 49.3% of patient visits and non-white minority groups at 41.4% of visits. For all racial/ethnic groups, x-ray imaging was the most commonly ordered modality followed in descending order by CT, ultrasound, and MRI. White patients had the highest image utilization rates for all imaging modalities with the exception of ultrasound which was most frequently ordered for Hispanic patients.

**Table 1:** Demographic characteristics from United States emergency department patient visits by race/ethnicity from 2006 to 2016

	Patient race and ethnicity					
	White vs Minority		Sub-divided Minority Groups			
	White	All minorities	Black	Hispanic	Asian/Other	*p*-value^b^
Count (weighted)	865,098,482	582,045,941	323,144,348	212,126,410	46,775,183	
Age (mean (SE))	40.5 (0.25)	32.2 (0.30)	33.6 (0.34)	29.3 (0.45)	34.8 (0.59)	< 0.001
Gender (% male)	45.5 (0.19)	44.5 (0.25)	43.1 (0.32)	46.3 (0.34)	46.1 (0.64)	< 0.001
Total number of chronic conditions ^a^ (mean (SE))	0.8 (0.03)	0.6 (0.02)	0.7 (0.03)	0.5 (0.03)	0.6 (0.04)	< 0.001
**Insurance status (% (SE)):**						< 0.001
Private insurance	34.1 (0.47)	23.4 (0.51)	22.8 (0.63)	22.1 (0.63)	33.0 (1.17)	
Medicare	21.6 (0.34)	11.2 (0.27)	12.9 (0.35)	8.3 (0.32)	12.5 (0.70)	
Medicaid	21.3 (0.49)	36.6 (0.71)	35.2 (0.71)	40.2 (1.11)	30.2 (1.29)	
Uninsured, unknown, other	23.0 (0.55)	28.8 (0.70)	29.1 (0.80)	29.5 (0.89)	24.3 (1.31)	
**Imaging ordered (% of all visits (SE)):**						
Any imaging exam	49.3 (0.48)	41.4 (0.44)	41.3 (0.62)	41.0 (0.49)	43.5 (1.02)	< 0.001
-X-ray	36.2 (0.38)	30.5 (0.38)	31.3 (0.54)	28.8 (0.39)	32.4 (0.95)	< 0.001
-CT	17.1 (0.34)	12.3 (0.25)	11.8 (0.34)	12.7 (0.33)	13.9 (0.55)	< 0.001
-MRI	0.7 (0.04)	0.6 (0.04)	0.5 (0.05)	0.6 (0.06)	0.9 (0.12)	< 0.001
-Ultrasound	3.5 (0.13)	3.9 (0.13)	3.5 (0.16)	4.6 (0.18)	3.8 (0.26)	< 0.001

*SE* standard error, *CT* computed tomography, *MRI* magnetic resonance imaging

Demographic characteristics of United States Emergency Department visits for the years 2006–2016 are tabulated by patient race/ethnicity. The counts in the first row represent the survey weighted numbers of patient visits in the data set over the study time period. The mean values and proportions in the body of the table are formulated using the survey weighted values to produce national level estimates

^a^ Data only available from 2012 to 2016

^b^ ANOVA test was used to test if the age and total number of chronic conditions were different across racial and ethnic groups. Pearson’s chi-squared test was used to compare the proportion of male, distribution of insurance status, and proportion of different types of imaging exams ordered across four racial/ethnic groups. Bonferroni calculation used for multiple comparison correction

**Fig. 1:**
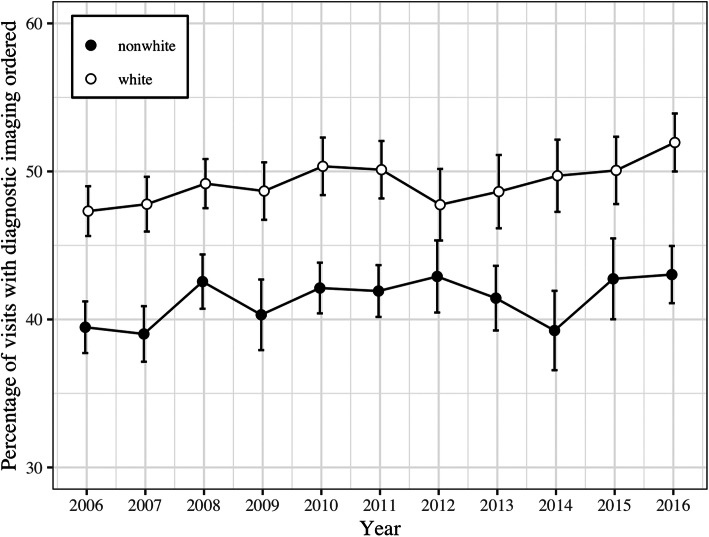
Proportion of patient encounters with diagnostic imaging ordered during United States Emergency Department visits from 2006 to 2016 by patient race/ethnicity, comparing white versus non-white minorities

**Fig. 2:**
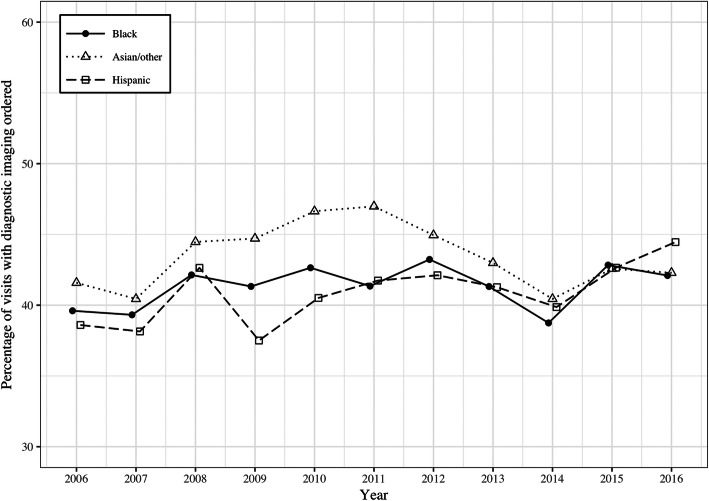
Proportion of patient encounters with diagnostic imaging ordered during United States Emergency Department visits from 2006 to 2016 by patient race/ethnicity, comparing non-white sub-groups. Error bars are omitted for clarity

The logistic regression model indicated that this association between image ordering and patient race/ethnicity held true even when controlling for a variety of other patient and hospital factors that might reasonably predict the ordering of an imaging exam. Table 2 shows the odds ratios for pair-wise comparison of minority racial groups compared to whites in the controlled regression model. The greatest difference in the odds exists between white patients and black, with the odds of a black patient having an imaging exam of any kind 22% less than the odds for a white patient. A significant disparity also existed between white patients and those categorized in the collapsed category of Asian/other. Hispanic patients were also less likely to receive imaging, but this comparison did not reach statistical significance in the pairwise comparison controlling for other hospital and patient characteristics. The differential use of medical imaging by patient race and ethnicity persisted when compared by imaging modality. White patients had the highest odds of receiving x-ray and CT exams. Patients in the category of Asian/other had the highest odds for MRI and Hispanic patients the highest odds for ultrasound however neither of these differences achieved significance in the controlled model. Black patients had the lowest odds of receiving imaging exams of any kind with the exception of ultrasound where patients in the Asian/other category showed slightly lower odds. The differences in diagnostic imaging rates between whites and minority groups remained significant across the 11-year study period with insufficient evidence to conclude that there was variation in these racial and ethnic differences in image ordering by year, an indicator that the disparate use of imaging did not change over time. Figures 1 and 2 illustrate the differential image utilization rates by patient race and ethnicity over the study time period, comparing whites versus non-white minorities and minority sub-groups respectively.

**Table 2:** Adjusted odds ratios with 95% confidence intervals for the effect of patient race/ethnicity on the likelihood of diagnostic imaging being ordered during United States emergency department visits from 2006 to 2016

	Patient race/ethnicity^a^				
Imaging modality:	White	Any minority group	Black	Hispanic	Asian/other
-Any imaging	1.00 (ref)	0.84 (0.79–0.89)	0.78 (0.72–0.84)	0.94 (0.89–1.00)	0.82 (0.72–0.94)
-X-ray	1.00 (ref)	0.91 (0.86–0.96)	0.89 (0.82–0.96)	0.93 (0.87–1.00)	0.93 (0.81–1.06)
-CT	1.00 (ref)	0.78 (0.73–0.84)	0.70 (0.64–0.76)	0.92 (0.84–1.01)	0.78 (0.67–0.92)
-MRI	1.00 (ref)	0.86 (0.65–1.13)	0.75 (0.54–1.05)	0.90 (0.62–1.29)	1.34 (0.88–2.04)
-Ultrasound	1.00 (ref)	1.03 (0.92–1.14)	0.99 (0.88–1.12)	1.10 (0.94–1.27)	0.91 (0.69–1.20)

Adjusted odds ratios evaluating the influence of patient race/ethnicity on the ordering of medical imaging during United States emergency department visits from 2006 to 2016 are shown. Odds ratios were adjusted for age, gender, insurance payor, number of co-morbid conditions, hospital region and setting, and survey year

^a^The collapsed racial ethnic categories of non-Hispanic white, non-Hispanic black, Hispanic, and Asian/other were used for analysis

A variety of other factors included in the regression analysis also had a significant association with the likelihood of imaging being ordered. These are summarized in Table 3. Holding the other variables constant, diagnostic imaging rates were significantly higher with advancing age, with a greater number of comorbidities, in male patients, and in urban hospitals. Rate of imaging varied by geographic region with the highest odds of imaging utilization in Midwestern and Southern hospitals although this reached statistical significance only in the pairwise comparison between the Southern and Western regions. There was insufficient evidence to suggest that the overall rate of imaging in the ED varied by year.

**Table 3:** Adjusted odds ratios of patient and hospital factors on the likelihood of diagnostic imaging being ordered during United States Emergency Department visits from 2006 to 2016

Patient or hospital factor	Odds Ratio (95% CI)
Age	1.02 (1.01, 1.02)
Gender (female vs male)	0.95 (0.91, 0.99)
Total chronic conditions^a^	1.12 (1.09, 1.15)
Urban vs non-urban^b^	1.24 (1.12, 1.38)
Midwest vs West	1.11 (0.97, 1.27)
Northeast vs West	0.94 (0.81, 1.10)
South vs West	1.16 (1.04, 1.29)
Medicaid vs Private	0.82 (0.76, 0.88)
Medicare vs Private	0.87 (0.80, 0.95)
Uninsured/other vs Private	0.82 (0.76, 0.89)

The influence of multiple patient and hospital factors on the likelihood of any imaging exam being ordered during United States Emergency Department encounters is shown with adjusted odds ratios from the logistic regression model. Model variables include patient race/ethnicity, age, gender, insurance payor, number of co-morbid conditions, hospital region and setting, and survey year

^a^ Data only available from 2012 to 2016

^b^ Urban hospitals are designated as in a Metropolitan Statistical Area (MSA) by the NHAMCS survey

## Discussion

This analysis of a large nationally representative database of ED visits over an 11-year period demonstrates a strong and consistent association with patient race/ethnicity and the likelihood of diagnostic imaging use. This trend, in which patients in minority racial and ethnic groups were less likely to receive an imaging examination, persisted even when controlling for measurable confounders and remained true throughout the study time period. Our results raise strong concern that minority patients face a consistent pattern of systematic bias influencing diagnostic decision-making during ED visits. Imaging was ordered in 46% of all ED encounters in our analysis testifying to the key role it plays in the work up for many clinical scenarios. The systematic underutilization of imaging in racial and ethnic minorities may therefore place these groups at risk for delayed or incorrect diagnosis, potentially leading to poorer outcomes. This adds to the body of previous work demonstrating worsened outcomes in minorities and may indicate a potential step in the causal pathway, specifically that the threshold to obtain diagnostic imaging may be higher in minorities than for white patients. Although not well investigated specifically in the ED setting, delays or barriers to appropriate imaging can have unpredictable but negative downstream effects including diagnosis of disease at a more advanced stage [32], longer hospital length of stay [33], or unnecessary hospital admission [34].

Although the dominant trend was for higher rates of imaging in white patients, this varied slightly by imaging modality. Specifically, in contrast to the other imaging modalities, white patients were slightly less likely to receive ultrasound exams compared to non-white patients (3.5% of visits compared to 3.9% of visits respectively). Additionally, the highest rate of MRI utilization was for patients in the combined racial/ethnic category of Asian and other. However, the absolute difference was small (0.9% of visits for Asian/other and 0.5–0.7% in the other patient groups) and did not achieve significance in the controlled model. The reasons for these small variations are unknown but it should be noted that ultrasound and MRI were utilized in the ED setting much less commonly than x-ray or CT exams where minority groups clearly lagged in imaging utilization rates. Nonetheless, this variation bears further study, perhaps at the local level where more detailed individual patient information is available.

It is important to note that more imaging does not always equate to better health care, and it is possible that to some extent the higher rate of imaging use in white patients reflects over-utilization. Examples of race-based over-treatment include the increased rate of antibiotic use for viral illnesses in white patients [35] and increased rates of *Clostridium difficile* infection associated with better access to the health care system [36]. However, imaging over-utilization is unlikely to explain the large and consistent differences in imaging rates between racial and ethnic groups, and it is doubtful that the lower rate of imaging utilization in minorities reflects best available care. Nonetheless, further research examining the influence of patient race/ethnicity on image ordering patterns in more specifically designated clinical scenarios may help to further elucidate these trends and their implications.

This study does have several important limitations. First, patient race and ethnicity are classified by hospital and survey staff rather than by patient self-identification creating potential for misclassification. It seems unlikely that this would alter the study conclusions as perceived patient race/ethnicity may be as or more important than self-identification in a study on the potential for ordering provider implicit bias. However, the extent to which misclassification of racial and ethnic identity occurs within the NHAMCS dataset is unknown as is its effect on the observed effects of race and imaging use in our study. As a separate concern, although the use of the NHAMCS dataset for analysis provides a national overview, the level of detail for any individual patient encounter is limited. The total number of patient co-morbidities is listed but detailed patient level data is not available so the appropriateness of imaging for any individual patient cannot be ascertained. A large body of previous research highlights a range of health conditions which disproportionately affect people of color [37] suggesting that the decreased use of imaging in minority groups may reflect inadequate patient care, but as not all health conditions require medical imaging in the acute care setting, further research evaluating differences in imaging use for more specific, evidence-based indications is necessary.

The data used in this study are cross-sectional and thus patient outcomes over time are unknown. Whether the decreased use of imaging in minority groups results in worse outcomes cannot be directly answered by this study. Nonetheless, there is reason for concern that this may be the case. For example, minority groups are frequently diagnosed at later stages of cancer than white patients [32, 38, 39] and minority pediatric patients with appendicitis are more likely to progress to perforation and abscess than whites requiring longer hospitalizations and more frequent ICU admissions [40]. Both scenarios may reflect a higher threshold to obtain imaging for minority patients with associated health consequences.

An additional limitation is the possibility of residual confounding. Not every patient and hospital characteristic that may affect the decision to order imaging is available in the data set. Perhaps most importantly, there is no direct measurement of patient socioeconomic status. Racial disparities in health care may decrease when controlled for socioeconomic standing [4, 5]. Patient insurance payor served as an imperfect surrogate marker for socioeconomic standing in this study and racial disparity persisted despite controlling for this variable as well as other key possible confounders such as the number of patient co-morbidities. Nonetheless, it is possible that some of the variability in imaging utilization is related to un-measured confounders of disease severity or socio-economic status which would lead to an overestimation of the effect of patient race and ethnicity on imaging use. Although the possibility of residual confounding should always be considered, the strength and consistency of the findings across a nation-wide database over an 11-year time period make it unlikely that racial and ethnic differences in imaging utilization are explained entirely by unknown confounders.

Although our analysis reflects a consistent difference in image ordering rates based on patient race and ethnicity, the reasons for this are likely complex. Undoubtably individual health care providers have a variable threshold for the decision to use diagnostic imaging during any given patient encounter. This may be based on explicit criteria such as local practice protocols, patient presenting symptoms, vital signs, co-morbidities, and age or may reflect less measurable factors such as the physician’s clinical instinct and judgement. The interplay of race and ethnicity may affect the patient/physician relationship in multiple ways, tipping the balance of diagnostic decision making. This may be a result of the well-known phenomenon of implicit/unconscious bias [41–43] or may be more indirect resulting from other identifiable impacts of racial and ethnic identity on the patient/provider relationship including levels of trust, willingness to communicate, and perceived cultural differences [44–46]. Strategies used to overcome the disparities observed in our study must account for these potential pathways.

A variety of other factors included in our analysis also demonstrated a significant association with diagnostic imaging use each of which bears further investigation. Predictably, advancing age and increasing number of co-morbidities was associated with higher imaging use. Imaging also varied significantly by patient gender with male patients receiving more imaging exams than females. Much of this may be explainable by a greater provider inclination to avoid tests with ionizing radiation (x-ray and CT) in females, particularly of child-bearing age. However, the possibility of gender bias should also be considered. Gender-based health care disparities have been found by investigators in other contexts. This includes critical differences in chest pain management in ambulatory care in men vs. women, with men being 2.5 times more likely to be referred to a cardiologist than women [47]. Gender bias in health care has been well documented, [48–50], but further investigation of gender differences in the use of diagnostic imaging is warranted with more specifically defined clinical scenarios before firm conclusions can be drawn. The differences in imaging rates between designated urban and non-urban hospitals and between different regions of the country are also important but less easily interpreted as there is no defined “ideal” imaging rate and this may represent either under or over utilization of imaging resources. Further studies with measures of quality imaging use may better define these regional differences.

## Conclusion

The results of our study indicate that despite many years of study and intervention, racial disparities may remain a part of our health care system. Further studies are needed to better define these disparities in more specific clinical settings, and an emphasis should be placed on identifying evidence-based strategies to mitigate them and pursue an agenda of health equity.

## Data Availability

The datasets analyzed during the current study are available in the National Center for Health Statistics repository available at https://www.cdc.gov/nchs/ahcd/rdc_data.htm.

## Abbreviations

ED: Emergency Department
CT: Computed Tomography
MRI: Magnetic Resonance Imaging
NHAMCS: National Hospital Ambulatory Medical Care Survey

## References

[1] Health, United States, 2015: With Special Feature on Racial and Ethnic Health Disparities. Health, United States. Hyattsville (MD)2016. 27308685

[2] U.S. Department of Health and Human Services. 2016 National Healthcare Quality and Disparities Report. (Publication No. 17–0001). Maryland, MD: Agency for Healthcare Research and Quality; 2017.

[3] Staff IoM (2004). Unequal treatment: confronting racial and ethnic disparities in healthcare: National Academies Press.

[4] Williams DR, Wyatt R (2015). Racial bias in health care and health: challenges and opportunities. Jama..

[5] Williams DR, Mohammed SA, Leavell J, Collins C (2010). Race, socioeconomic status, and health: complexities, ongoing challenges, and research opportunities. Ann N Y Acad Sci.

[6] Fiscella K, Sanders MR (2016). Racial and ethnic disparities in the quality of health care. Annu Rev Public Health.

[7] Ayanian JZ, Landon BE, Newhouse JP, Zaslavsky AM (2014). Racial and ethnic disparities among enrollees in Medicare advantage plans. N Engl J Med.

[8] Quintana JM, Goldmann D, Homer C (1997). Social disparities in the use of diagnostic tests for children with gastroenteritis. Int J Qual Health Care.

[9] Bazarian JJ, Pope C, McClung J, Cheng YT, Flesher W (2003). Ethnic and racial disparities in emergency department care for mild traumatic brain injury. Acad Emerg Med.

[10] Wells KJ, Roetzheim RG (2007). Health disparities in receipt of screening mammography in Latinas: a critical review of recent literature. Cancer Control.

[11] Ford ES, Cooper RS (1995). Racial/ethnic differences in health care utilization of cardiovascular procedures: a review of the evidence. Health Serv Res.

[12] Carlisle DM, Leake BD, Shapiro MF (1997). Racial and ethnic disparities in the use of cardiovascular procedures: associations with type of health insurance. Am J Public Health.

[13] Fishman J, McLafferty S, Galanter W (2018). Does spatial access to primary care affect emergency department utilization for nonemergent conditions?. Health Serv Res.

[14] Parekh N, Jarlenski M, Kelley D (2018). Disparities in access to primary care and emergency department utilization in a large Medicaid program. J Health Disparities Res Practice.

[15] Johnson TJ, Hickey RW, Switzer GE, Miller E, Winger DG, Nguyen M (2016). The impact of cognitive stressors in the emergency department on physician implicit racial bias. Acad Emerg Med.

[16] Schrader CD, Lewis LM (2013). Racial disparity in emergency department triage. J Emergency Med.

[17] Richardson LD, Babcock Irvin C, Tamayo-Sarver JH (2003). Racial and ethnic disparities in the clinical practice of emergency medicine. Acad Emerg Med.

[18] Pletcher MJ, Kertesz SG, Kohn MA, Gonzales R (2008). Trends in opioid prescribing by race/ethnicity for patients seeking care in US emergency departments. JAMA..

[19] Heins JK, Heins A, Grammas M, Costello M, Huang K, Mishra S (2006). Disparities in analgesia and opioid prescribing practices for patients with musculoskeletal pain in the emergency department. J Emerg Nurs.

[20] Strakowski SM, Lonczak HS, Sax KW, West SA, Crist A, Mehta R, et al. The effects of race on diagnosis and disposition from a psychiatric emergency service. J Clin Psychiatry. 1995;56(3):101–7. 7883727

[21] Park CY, Lee MA, Epstein AJ (2009). Variation in emergency department wait times for children by race/ethnicity and payment source. Health Serv Res.

[22] Wippold FJ, Brown DC, Broderick DF, Burns J, Corey AS, Deshmukh TK (2015). ACR appropriateness criteria dementia and movement disorders. J Am Coll Radiol.

[23] Ross AB, Lee KS, Chang EY, Amini B, Bussell JK, Gorbachova T (2019). ACR appropriateness criteria® acute hip pain-suspected fracture. J Am Coll Radiol.

[24] Earls JP, Woodard PK, Abbara S, Akers SR, Araoz PA, Cummings K (2014). ACR appropriateness criteria asymptomatic patient at risk for coronary artery disease. J Am Coll Radiol.

[25] Schrager J, Patzer R, Kim J, Pitts S, Chokshi F, Phillips J, et al. Racial and Ethnic Differences in Diagnostic Imaging Utilization During Adult Emergency Department Visits in the United States, 2005 to 2014. J Am Coll Radiol 2019. doi: 10.1016/j.jacr.2019.03.002. Epub 2019/05/17. PubMed PMID: 31092354. 10.1016/j.jacr.2019.03.002 31092354

[26] National Center for Health Statistics - Ambulatory Health Care Data [5/17/2019]. Available from: https://www.cdc.gov/nchs/ahcd/index.htm. Accessed 29 Jan 2020.

[27] [8/25/2020]. Available from: https://www.healthypeople.gov/2020/data-source/national-hospital-ambulatory-medical-care-survey. Accessed 29 Jan 2020.

[28] NHAMCS Survey Instructions [5/17/2019]. Available from: https://www.cdc.gov/nchs/data/series/sr_01/sr01_034acc.pdf. Accessed 29 Jan 2020.

[29] Steyerberg EW, van Veen M (2007). Imputation is beneficial for handling missing data in predictive models. J Clin Epidemiol.

[30] Owens PL, Barrett ML, Gibson TB, Andrews RM, Weinick RM, Mutter RL (2010). Emergency department care in the United States: a profile of national data sources. Ann Emerg Med.

[31] Hostetler MA, Auinger P, Szilagyi PG (2002). Parenteral analgesic and sedative use among ED patients in the United States: combined results from the National Hospital Ambulatory Medical Care Survey (NHAMCS) 1992-1997. Am J Emerg Med.

[32] Iqbal J, Ginsburg O, Rochon PA, Sun P, Narod SA (2015). Differences in breast cancer stage at diagnosis and cancer-specific survival by race and ethnicity in the United States. Jama..

[33] Abujudeh HH, Kaewlai R, McMahon PM, Binder W, Novelline RA, Gazelle GS (2011). Abdominopelvic CT increases diagnostic certainty and guides management decisions: a prospective investigation of 584 patients in a large academic medical center. AJR Am J Roentgenol.

[34] Cournane S, Conway R, Creagh D, Byrne DG, Sheehy N, Silke B (2016). Radiology imaging delays as independent predictors of length of hospital stay for emergency medical admissions. Clin Radiol.

[35] Goyal MK, Johnson TJ, Chamberlain JM, Casper TC, Simmons T, Alessandrini EA, et al. Racial and Ethnic Differences in Antibiotic Use for Viral Illness in Emergency Departments. Pediatrics. 2017;140(4). doi: 10.1542/peds.2017-0203. Epub 2017/09/06. PubMed PMID: 28872046; PubMed Central PMCID: PMCPMC5613999 conflicts of interest to disclose. 10.1542/peds.2017-0203 PMC5613999 28872046

[36] Mao EJ, Kelly CR, Machan JT. Racial Differences in *Clostridium difficile* Infection Rates Are Attributable to Disparities in Health Care Access. Antimicrob Agents Chemother. 2015;59(10):6283–7. doi: 10.1128/AAC.00795-15. Epub 2015/08/08. PubMed PMID: 26248363; PubMed Central PMCID: PMCPMC4576108. 10.1128/AAC.00795-15 PMC4576108 26248363

[37] Meyer PA, Yoon PW, Kaufmann RB (2013). Introduction: CDC health disparities and inequalities report-United States, 2013. MMWR supplements.

[38] Tawk R, Abner A, Ashford A, Brown C (2016). Differences in colorectal cancer outcomes by race and insurance. Int J Environ Res Public Health.

[39] Virnig BA, Baxter NN, Habermann EB, Feldman RD, Bradley CJ (2009). A matter of race: early-versus late-stage cancer diagnosis. Health Aff.

[40] Wang L, Haberland C, Thurm C, Bhattacharya J, Park K (2015). Health outcomes in US children with abdominal pain at major emergency departments associated with race and socioeconomic status. PLoS One.

[41] Dehon E, Weiss N, Jones J, Faulconer W, Hinton E, Sterling S (2017). A systematic review of the impact of physician implicit racial Bias on clinical decision making. Acad Emerg Med.

[42] Sabin JA, Greenwald AG. The influence of implicit bias on treatment recommendations for 4 common pediatric conditions: pain, urinary tract infection, attention deficit hyperactivity disorder, and asthma. Am J Public Health. 2012;102(5):988–95. doi: 10.2105/AJPH.2011.300621. Epub 2012/03/17. PubMed PMID: 22420817; PubMed Central PMCID: PMCPMC3483921. 10.2105/AJPH.2011.300621 PMC3483921 22420817

[43] Kapur N (2015). Unconscious bias harms patients and staff. BMJ..

[44] Saha S, Arbelaez JJ, Cooper LA. Patient-physician relationships and racial disparities in the quality of health care. Am J Public Health. 2003;93(10):1713–9. doi: 10.2105/ajph.93.10.1713. Epub 2003/10/10. PubMed PMID: 14534227; PubMed Central PMCID: PMCPMC1448039. 10.2105/ajph.93.10.1713 PMC1448039 14534227

[45] Cooper LA, Beach MC, Johnson RL, Inui TS. Delving below the surface. Understanding how race and ethnicity influence relationships in health care. J Gen Intern Med. 2006;21 Suppl 1:S21–S27. doi: 10.1111/j.1525-1497.2006.00305.x. Epub 2006/01/13. PubMed PMID: 16405705; PubMed Central PMCID: PMCPMC1484840. 10.1111/j.1525-1497.2006.00305.x PMC1484840 16405705

[46] Dedier J, Penson R, Williams W, Lynch T (1999). Race, ethnicity, and the patient-caregiver relationship. Oncologist..

[47] Clerc Liaudat C, Vaucher P, De Francesco T, Jaunin-Stalder N, Herzig L, Verdon F, et al. Sex/gender bias in the management of chest pain in ambulatory care. Womens Health (Lond). 2018;14:1745506518805641. Epub 2018/10/30. doi: 10.1177/1745506518805641. PubMed PMID: 30370833; PubMed Central PMCID: PMCPMC6300868. 10.1177/1745506518805641 PMC6300868 30370833

[48] Rabinowitz LG (2018). Recognizing blind spots - a remedy for gender Bias in medicine?. N Engl J Med.

[49] Biddle C, Fallavollita JA, Homish GG, Orom H. Gender bias in clinical decision making emerges when patients with coronary heart disease symptoms also have psychological symptoms. Heart Lung. 2018. doi: 10.1016/j.hrtlng.2018.11.005. Epub 2019/01/01. PubMed PMID: 30595342. 10.1016/j.hrtlng.2018.11.005 30595342

[50] Daugherty SL, Blair IV, Havranek EP, Furniss A, Dickinson LM, Karimkhani E, et al. Implicit Gender Bias and the Use of Cardiovascular Tests Among Cardiologists. J Am Heart Assoc. 2017;6(12). doi: 10.1161/JAHA.117.006872. Epub 2017/12/01. PubMed PMID: 29187391; PubMed Central PMCID: PMCPMC5779009. 10.1161/JAHA.117.006872 PMC5779009 29187391

